# Alpha-synuclein measured in cerebrospinal fluid from patients with Alzheimer’s disease, mild cognitive impairment, or healthy controls: a two year follow-up study

**DOI:** 10.1186/s12883-016-0706-0

**Published:** 2016-09-21

**Authors:** Guro Berge, Sigrid B. Sando, Grethe Albrektsen, Camilla Lauridsen, Ina Møller, Gøril R. Grøntvedt, Geir Bråthen, Linda R. White

**Affiliations:** 1 Department of Neuroscience, Faculty of Medicine, Norwegian University of Science and Technology (NTNU), Trondheim, NO 7491 Norway; 2 Department of Neurology, University Hospital of Trondheim, Trondheim, Norway; 3 Department of Public Health and General Practice, Faculty of Medicine, Norwegian University of Science and Technology (NTNU), Trondheim, Norway

**Keywords:** Longitudinal, Biomarker, Dementia, ELISA

## Abstract

**Background:**

α-Synuclein has been proposed as a potential biomarker for Alzheimer’s disease (AD) and amnestic mild cognitive impairment (aMCI). However, results from α-synuclein measurements in cerebrospinal fluid (CSF) have been inconclusive, and to our knowledge, longitudinal studies of changes prior to the AD diagnosis have not been investigated.

**Methods:**

Levels of α-synuclein at baseline and after one and two years were measured in CSF, by enzyme-linked immunosorbent assay. Twenty-six patients with early AD (AD-AD), 48 patients with aMCI, subdivided as 23 that developed AD during follow-up (MCI-AD), and 25 that did not (MCI-MCI), and 25 healthy control individuals, were included. One-way ANOVA was applied to compare mean α-synuclein baseline values between all four study groups, and a linear mixed model was used to compare mean change over time between the three patient groups. Linear associations between α-synuclein and amyloid-β 1–42 (Aβ42), amyloid-β 1–40 (Aβ40), total tau and phosphorylated tau were also examined.

**Results:**

A large variation in individual α-synuclein CSF levels was observed, particularly in the MCI-AD group. No significant differences were found in mean α-synuclein levels between all the study groups at baseline. When using a linear mixed model, no significant differences were found at follow-up for estimated mean changes between the patient groups. MCI-AD patients with short duration of symptoms prior to inclusion in the study (≤2 years) had considerably higher mean CSF α-synuclein levels compared to patients with a longer symptom duration (802.2 vs. 442.8 pg/mL, *p =* 0.01). No such difference was seen in the MCI-MCI or AD-AD groups. Significant linear associations (*p <* 0.0005) between α-synuclein and Aβ40, total tau and phosphorylated tau were found.

**Conclusion:**

The observed difference in mean CSF α-synuclein level according to duration of symptoms in the MCI-AD group, may be an indication of changes related to disease progression. However, the lack of significant differences between groups, as well as the large individual variation in CSF levels of α-synuclein in the present study, suggest that α-synuclein is not a useful biomarker for AD.

**Electronic supplementary material:**

The online version of this article (doi:10.1186/s12883-016-0706-0) contains supplementary material, which is available to authorized users.

## Background

Alzheimer’s disease (AD) is the most common cause of dementia, and is considered to be a slow progressive heterogeneous disorder resulting in death of neurons and progressive cognitive decline. The disease is preceded by a prodromal phase, mild cognitive impairment, which is usually amnestic (aMCI) [1]. The ability to diagnose patients at an early stage before dementia develops is urgent.

Classical AD neuropathology demonstrates amyloid plaques and neurofibrillary tangles [2, 3], and cerebrospinal fluid (CSF) levels of amyloid-β 1–42 (Aβ42), total tau (t-tau), and phosphorylated tau (p-tau) are promising diagnostic biomarkers. Patients with AD usually have lower CSF levels of Aβ42, and higher levels of tau compared to healthy controls. Measurement of these biomarkers in CSF is included in new suggested criteria as complementary to a clinical diagnosis [4]. The most abundant amyloid form, Aβ40, does not distinguish as well between patients with AD and healthy control individuals [5, 6].

In addition to plaques and tangles, up to 60 % of patients with AD also show evidence of Lewy body pathology [7–11], traditionally considered a hallmark of synucleinopathies such as Parkinson’s disease and dementia with Lewy bodies [12]. The presynaptic α-synuclein protein [13] is the major constituent of Lewy bodies and Lewy neurites [14], and is found in CSF at measurable levels [15, 16]. In most studies, patients with AD or aMCI show increased CSF levels of α-synuclein compared to healthy controls [17–21], perhaps due to release from damaged neuronal cell bodies and other processes during neurodegeneration [17]. However, no difference [22, 23], or a reduced level [24] have also been found. In CSF from patients with AD α-synuclein has shown strong correlation with t-tau and p-tau in some studies [19–21], but not all [25]. Moreover, an interdependent effect between α-synuclein, tau and amyloid-β in neurodegeneration has been proposed [26]. Results from experimental research on transgenic DLB-AD mice have indicated that α-synuclein, tau and amyloid-β proteins may act synergistically by mutually accelerating their accumulation and aggregation, leading to rapid cognitive decline [27].

α-Synuclein has been proposed as an additional CSF biomarker for AD and aMCI [17, 20]. To our knowledge, no previous studies have investigated changes in α-synuclein levels in CSF during the time window when the disease progresses from aMCI to AD. The aim of the present study was to evaluate the diagnostic potential of CSF levels of α-synuclein in the early stages of AD. CSF levels of α-synuclein were measured in patients with aMCI at the time of inclusion and during a two-year follow-up period with and without progression to AD, as well as in patients with AD from baseline and during two years of follow-up, and in healthy control individuals (baseline levels only). A potential association between α-synuclein and the *APOE* ɛ4 allele (the greatest genetic risk factor for AD [28]) was investigated, as well as potential correlations between α-synuclein and other known biomarkers for AD.

## Method

### Study participants

The present study included a total of 74 patients and 25 controls enrolled in a two-year follow-up study from the summer of 2009. All subjects were ethnic Norwegians between 53 and 78 years of age and recruited through the Department of Neurology, University Hospital of Trondheim, by two neurologists (SBS or GRG). Neurological examination was performed on both patient and control groups, including the Mini Mental State Examination (MMSE) [29] and the Ten-Word Test with delayed recall as taken from ADAS Cog [30], as well as cerebral MRI at 3 T at baseline and after two years. *APOE* genotyping was performed on blood samples according to the method described elsewhere [31]. General exclusion criteria were insufficient sight and hearing to complete the cognitive testing, a present psychiatric or malignant disease, use of anti-coagulating medication, or high alcohol consumption.

### Patients diagnosed with aMCI or AD

At inclusion 26 patients were diagnosed with early AD (National Institute of Neurological and Communicative Disorders and Stroke and the Alzheimer’s Disease and Related Disorders Association (NINCDS-ADRDA) criteria [32]), and 48 with aMCI (The International Working Group on Mild Cognitive Impairment Criteria [33]). Patients returned to the hospital one and two years after inclusion, with lumbar puncture and neuropsychological tests performed on each occasion. A few patients missed certain time-points of lumbar puncture. During follow-up, 23 of the aMCI patients kept their initial diagnosis (designated MCI-MCI), while 25 developed AD (designated MCI-AD). Patients diagnosed with AD from baseline comprised the AD-AD group. These three patient groups were compared in the statistical analyses. A detailed overview of the clinical work-up is described elsewhere [34].

### Healthy controls

Twenty-five healthy elderly volunteers were recruited as controls from societies for retired people in central Norway, or caregivers not genetically related to the patients. Lumbar puncture was performed at inclusion, whereas neuropsychological examination was performed at both inclusion and after two years. Control individuals were assessed as being healthy for their age without signs of neurological disorders, and without first-degree relatives with dementia.

### Sampling and analysis of CSF

Patients and healthy controls underwent lumbar puncture, usually at the L4/L5 intervertebral space, and CSF samples were obtained as described previously [34]. Briefly, 1 mL aliquots of CSF were collected directly into Corning 2 mL polypropylene cryovials immersed in ice-water, and frozen within 30 min of lumbar puncture and stored at −80 °C [35, 36]. Only three CSF samples from patients were centrifuged prior to freezing due to contamination from blood. Erythrocyte counts in the remaining samples were (mean ± SD) 1.6 ± 2.8/μL (overall range 0–17 erythrocytes/μL). The albumin quotient (Q_A_ = albumin in CSF/albumin in plasma) [37] was calculated for all samples. All samples were transferred to −20 °C approximately one week prior to analysis, and were slowly thawed in ice-water on the morning of analysis. The CSF samples were analyzed by commercially-available enzyme-linked immunosorbent assay (ELISA) monoplex kits; α-synuclein (Invitrogen: cat.no KHB0061), Aβ1-42 (Innogenetics: Aβ42, cat. no. 80324), total tau protein (t-tau, cat. no. 80323), 181-phosphorylated tau (p-tau, cat. no. 80317), and Aβ-40 (IBL International: cat. no. 81015). All assays were run according to the manufacturers’ instructions. Patient and healthy control samples were run on the same plates, in duplicate and averaged. An internal control was run on five out of seven α-synuclein ELISA plates, giving an inter-assay CV of 10.2 %, while the intra-assay CV was 2.9 % (*n =* 7). CV values for the core biomarkers are given elsewhere [34], but biomarker results were unknown at the time of clinical diagnosis. None of the individuals were excluded from the study due to increased hemoglobin levels in the samples, which is known to be a confounding factor in α-synuclein quantification in CSF, resulting in artificially increased values [18].

### Statistical analyses

The four study groups (AD-AD, MCI-AD, MCI-MCI, healthy control individuals) were compared with respect to categorical demographic and clinical characteristics at the time of inclusion by means of the chi-square tests (overall and pairwaise comparisons). One-way ANOVA was conducted to compare characteristics measured on a continuous scale, and also mean baseline values of α-synuclein, Aβ42, Aβ40, t-tau and p-tau. In pairwise comparison between groups, the least significant difference (LSD) procedure was applied, adjusting the nominal significance level to 1 %. A linear mixed model with subject as random factor, and with interaction terms added, was applied to compare mean changes in α-synuclein levels during follow-up (measurements at inclusion, and after 1 and 2 years, respectively) between the three patients groups. Both non-linear (categorical) and linear (per year) time patterns were considered. Moreover, linear associations between α-synuclein and other biomarkers (Aβ42, Aβ40, t-tau, p-tau) were examined with Pearson’s correlation coefficient and linear regression analysis. The determination coefficient (R^2^) is reported as the measure of percent explained variance in the linear regression model.

## Results

Demographic and clinical characteristics, and core biomarkers at time of inclusion as well as *APOE* ɛ4 allele data are shown in Table 1. The study groups did not differ significantly in gender, mean age at inclusion, mean age at onset, or mean duration since the first symptom for the respective diagnosis. The AD-AD and the MCI-AD groups had a larger proportion of subjects with MMSE test scores below 27 compared to healthy controls (*p* ≤ 0.010). The patient groups also differed significantly; patients in the AD-AD group more often had a score below 27 compared with the MCI-MCI or MCI-AD groups, and a larger proportion of the patients in the MCI-AD group had a low score compared with patients in the MCI-MCI group. Similarly, all the patient groups had reduced delayed recall for the Ten-word Test (all *p <* 0.0005) compared with healthy control individuals, with the AD-AD and MCI-AD groups having poorer recall than the MCI-MCI group (both *p* ≤ 0.001). The proportion of *APOE* ε4 allele carriers differed significantly between the study groups (*p <* 0.0005), with the patients having considerably higher proportions of carriers than healthy control individuals.

**Table 1:** Demographic and clinical characteristics at inclusion in the study

Characteristics	AD-AD 26	MCI-AD 25	MCI-MCI 23	CTR 25	*p*-value
Gender, *n* (%)					
Female	13 (50.0)	14 (56.0)	10 (43.5)	15 (60.0)	
Male	13 (50.0)	11 (44.0)	13 (56.5)	10 (40.0)	0.68
Age at inclusion					
(mean ± SD)	64.2 ± 6.2	63.7 ± 4.4	65.4 ± 6.1	67.3 ± 5.0	0.10
Age at onset					
(mean ± SD)	61.3 ± 6.3	61.0 ± 4.5	62. 9 ± 6.7	na	0.50
Duration of symptoms					
(mean ± SD)	3.0 ± 1.8	2.8 ± 1.0	2.9 ± 2.3	na	0.94
Ten Word Test (delayed recall)					
(mean ± SD)	1.6 ± 1.6	2.1 ± 1.8	3.8 ± 1.8	7.0 ± 1.7	<0.0005
MMSE, *n* (%)					
< 27	24 (92.7)	12 (48.0)	4 (17.4)	0 (0)	
≥ 27	2 (7.7)	13 (52.0 )	19 (82.6)	25 (100.0)	<0.0005
*APOE* ɛ4 carrier					
*n* (%)	21 (80.8)	19 (76.0)	12 (54.5)	7 (28.0)	<0.0005
Aβ42 (pg/mL)	498.7 ± 208.5	535.0 ± 176.3	647.7 ± 344.5	1080.5 ± 266.1	<0.0005
Aβ40 (pg/mL)	15265 ± 5386	15003 ± 4453	14216 ± 8073	18207 ± 6595	0.24
T-tau (pg/mL)	769.0 ± 405.4	733.7 ± 515.5	369.0 ± 345.6	297.9 ± 129.9	<0.0005
P-tau (pg/mL)	96.6 ± 35.6	90.6 ± 33.8	60.1 ± 27.8	57.0 ± 16.8	<0.0005

*AD-AD* patients diagnosed with Alzheimer’s disease at inclusion, *MCI-AD* patients with amnestic mild cognitive impairment at inclusion, progressing to AD during 2 years of follow-up, *MCI-MCI* patients with MCI at inclusion keeping their initial diagnosis during 2 years of follow-up, *CTR* healthy control individuals

*MMSE* mini mental state examination, *Ten-Word Test* from ADAS Cog

*T-tau* total tau, *P-tau* phosphorylated tau

*APOE* genotype missing for one MCI-MCI patient

*Na* not applicable

Mean baseline concentrations of Aβ42, t-tau and p-tau measured in CSF differed significantly between the four groups (all *p <* 0.0005), whereas no significant difference in Aβ40 was found (*p =* 0.24). Aβ42 concentration levels were lower in all patient groups compared to control individuals (all *p <* 0.0005), while no significant differences were found when comparing the patient groups against each other. A significantly higher mean level of t-tau and p-tau was found in the MCI-AD and AD-AD groups compared to healthy controls (all *p <* 0.0005), as well as compared to MCI-MCI (all *p* ≤ 0.003). No significant difference in these biomarkers was found between the AD-AD and MCI-AD groups.

### α-Synuclein levels in CSF at baseline, and during follow-up of patients with AD or aMCI

One patient from the MCI-AD group had a considerably elevated CSF α-synuclein concentration at baseline, and another at two years (though both were in the range of the kit). The albumin quotient for these two patients (and patients in general), did not indicate a disturbance of the blood–brain barrier [37] (data not shown). The analysis of changes in α-synuclein was first carried out with the two extreme values excluded. Mean baseline concentrations and standard deviations of α-synuclein in all study groups, and mean values after 1 and 2 years in the three patient groups are shown in Table 2a. The highest baseline mean level of α-synuclein measured in CSF was found in the MCI-AD group, with the other groups having approximately similar mean levels, lowest for the AD-AD group (*p =* 0.26, test for overall difference between groups). The individual variation was large in all study groups, but the standard deviation was larger in the two MCI groups than in the AD-AD and the control groups (Table 2a). The difference in mean α-synuclein baseline level became more pronounced when the two extreme values were included in the analysis (Additional file 1: Table S1a). The overall test for difference between the four study groups was then closer to statistical significance (*p =* 0.08), and the test for difference between the MCI-AD and the AD-AD groups was significant at the 5 % level, but not at the 1 % level (*p =* 0.019).

**Table 2:** a) Observed group mean values (± SD) for α-synuclein at inclusion (baseline), and after one and two years, and b) predicted mean value (95 % CI) at baseline, together with estimated mean change (95 % CI) from baseline values^a^

	α-synuclein (pg/mL)					
	AD-AD	MCI-AD	MCI-MCI	CTR	*p*-value^b^	*p*-value^c^
a)						
Baseline	494.4 ± 275.0	671.8 ± 383.1	568.6 ± 390.5	514.4 ± 263.1	0.26	
1 year	466.0 ± 324.8	650.9 ± 458.4	559.5 ± 339.6	na		
2 years	439.4 ± 285.5	587.4 ± 386.5	565.7 ± 393.3	na		
b)						
Baseline	477.3 (333.3,621.4)	661.7 (514.3,809.0)	568.6 (415.4,721.2)		0.22	
Mean change						0.93
After 1 year	−11.3 (−76.9, 54.3)	−16.7 (−85.8, 52.3)	−9.0 (−77.8, 59.7)		0.23	
After 2 years	−9.0 (−77.7, 59.7)	−42.0 (−112.2, 28.1)	0.02 (−69.9, 69.9)		0.33	
Linear trend^d^	−4.6 (−38.6, 29.4)	−21.0 (−55.7, 13.7)	−0.09 (−34.6, 34.5)			0.67

*Na* not applicable

^a^Results based on linear mixed model with subject as random factor (two extreme values excluded)

^b^F-test for overall difference in mean α-synuclein levels between study groups (one-way ANOVA), or between patient groups (LMM)

^c^F-test for heterogeneity in mean change over time in α-synuclein between patient groups (categorical and linear interaction, respectively)

^d^Mean change per year, based on model with linear time trend

**Fig. 1:**
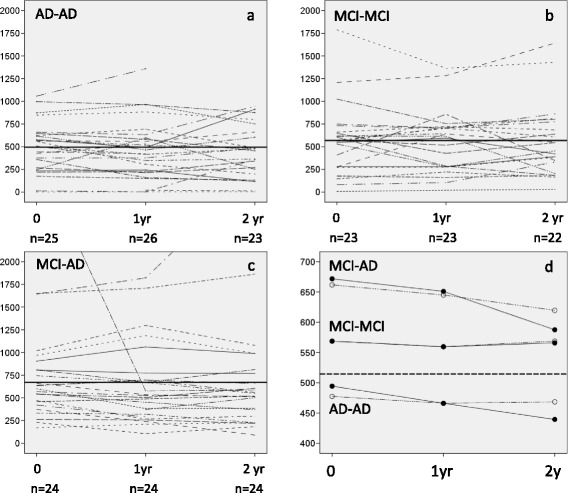
**a**-**c**: Individual α-synuclein levels (x-axis: pg/mL) in CSF over time in each patient group. The group mean value at inclusion (one extreme value excluded in the MCI-AD group) is indicated by a bold line. **d**: mean values, observed (filled circle) and predicted (open circle), of α-synuclein levels at each time point (two extreme values excluded, one at baseline and one after 2 years). The mean value for controls (baseline only) is shown as a dashed line

Individual time patterns of α-synuclein levels in the three patient groups are shown in Fig. 1a-c. Most patients had levels between 200–750 pg/mL. Except for one patient in the AD-AD group, values above 1000 pg/mL occurred only in the MCI-MCI and MCI-AD groups. A decrease in α-synuclein during the first year of follow-up was seen in all groups (extreme values excluded), but was more pronounced in the MCI-AD group than in the AD-AD and MCI-MCI groups (Table 2b, mean reduction of 16.7 vs. 11.4 and 9.0 pg/mL, respectively). In the MCI-AD group, α-synuclein values decreased also in the second year, in contrast to the pattern seen in the AD-AD and MCI-MCI groups (Table 2b). However, no significant changes in α-synuclein over time were seen within any of the groups, and the mean reduction over time did not differ between the groups (*p =* 0.93 and *p =* 0.67, test for categorical and linear interaction, respectively). Moreover, the mean α-synuclein levels did not differ significantly between the groups either after one year (*p =* 0.23) or after two years (*p =* 0.33). The mean α-synuclein values predicted on the basis of the linear mixed model fitted observed data rather well (Fig. 1d). In the analyses with the two extreme values included, the mean reduction in α-synuclein levels over time in the MCI-AD group was considerably more pronounced (Additional file 1: Table S1b). Nevertheless, no significant differences were found, either within or between the three patient groups.

### Mean α-synuclein CSF levels and duration of symptoms

Patients were included in the present study between one and seven years after onset of the first symptom related to aMCI or AD. In view of the large variation in individual α-synuclein levels at baseline, the mean α-synuclein level at time of inclusion in the study was compared between groups defined by duration of symptoms prior to inclusion (1–2 years vs. ≥ 3 years). In the MCI-AD group, those with the shortest duration of symptoms had significantly higher mean α-synuclein CSF concentration (802.2 pg/mL, confidence interval: 553.9 - 1051.5 pg/mL) than patients in the group with longer duration (442.8 pg/mL, confidence interval: 320.3 - 565.4 pg/mL), two sample *t*-test, *p =* 0.01, extreme value at baseline excluded. No significant differences were found according to duration of symptoms in the MCI-MCI or AD-AD groups.

### Association between α-synuclein and the core AD biomarkers

**Fig. 2:**
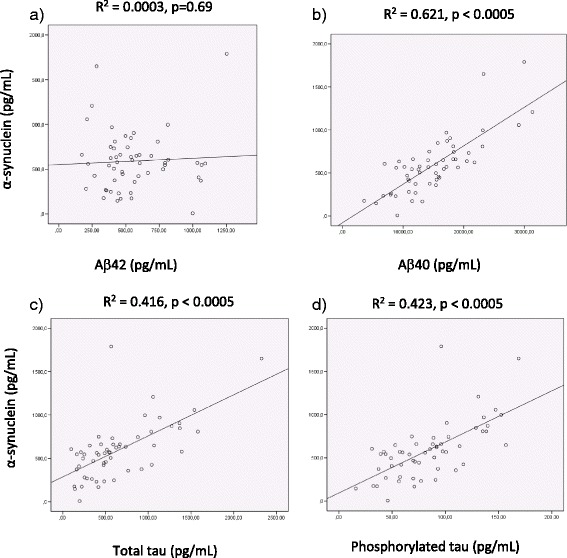
Scatterplots and line of best fit illustrating the association between α-synuclein and (**a**) Aβ42, (**b**) Aβ40, (**c**) total tau or (**d**) phosphorylated tau measured in CSF. Points represent the total patient study material at inclusion, extreme value excluded

In CSF samples from healthy control individuals, Aβ40, t-tau and p-tau all showed strong linear associations with CSF α-synuclein (determination coefficient R^2^ in range 57.3-61.8 %, *p <* 0.0005) whereas the association with Aβ42 was weaker (R^2^ of 24.7 %). In a combined analysis of all patient groups, the association between baseline CSF α-synuclein and Aβ40 was similar to that observed in healthy individuals (Fig. 2b), whereas the strength of linear association with t-tau and p-tau was slightly weaker (Fig. 2c and d, R^2^ = 41.6 % and 42.3 %, respectively, *p <* 0.0005). No significant association was found between Aβ42 and α-synuclein (*p =* 0.69), Fig. 2a. In a model with mutual adjustment for all these biomarkers, all factors together explained 67.3 % of the variation in α-synuclein, but only Aβ40 and p-tau contributed significantly. The direction of linear association between each biomarker and α-synuclein was consistently observed within all four study groups, except that Aβ42 was negatively correlated in the MCI-AD group (Pearson’s correlation coefficient −0.325) and positively correlated in the other study groups (Pearson’s correlation coefficient in range 0.075 – 0.499). The opposite direction of association with Aβ42 in the patient groups probably explains the lack of association in combined analysis of all patients.

### Associations between α-synuclein and APOE ɛ4

The mean concentration of α-synuclein did not differ significantly between *APOE* ɛ4 carriers and non-carriers in any group (Additional file 2 Table S2), though a more pronounced difference was detected in the MCI-AD group. In this patient group, carriers of *APOE* ɛ4 had a mean CSF α-synuclein level of 887.1 ± 692.9 pg/mL whereas non-carriers had a mean level of 442.3 ± 181.1 pg/mL. However, the difference was not statistically significant.

## Discussion

To our knowledge this is the first study to investigate CSF levels of α-synuclein during the transition from aMCI to AD. Patients with aMCI or AD showed comparatively stable levels of α-synuclein from inclusion and over a two year period. Except for a slightly higher baseline concentration of α-synuclein in the MCI-AD group, no differences were observed, either between the patient groups, or in comparisons with healthy control individuals. Although some measured levels were high, albumin quotients did not suggest leakage of α-synuclein from blood as being responsible for these high levels. Both the observed levels of α-synuclein, and lack of association with the *APOE* ɛ4 allele are in accordance with previous findings [17, 19, 21, 23]. Our results do not indicate marked differences in mean α-synuclein concentrations between the four groups, and the considerable variation in measured levels makes it difficult to find subtle differences between groups in a small material.

In our study, patient levels of α-synuclein did not differ to those found in healthy control individuals. This contrasts with numerous studies of the ‘core’ biomarkers, which usually vary markedly between controls and patients with AD, and MCI to some extent. However, we observed correlations between α-synuclein and t-tau or p-tau among patients and controls, as reported previously [20, 21]. A (weak) correlation between α-synuclein and Aβ42 was only found in the control group, perhaps because both substances have physiological synaptic functions [13, 38], which may have been disturbed during the disease process. However, the correlation between α-synuclein and Aβ40, was stronger than for other biomarkers. This peptide is less frequently analyzed in CSF, and the present observation in patients with AD is possibly a novel finding. Interestingly, this correlation has also been found recently between patients with Parkinson’s disease and healthy control individuals [39]. Aβ40 in CSF has been found to correlate with rapid cognitive decline [5], as has α-synuclein [17], potentially explaining the strong correlation between them.

The present finding of a higher mean α-synuclein CSF level at baseline in the MCI-AD group with shortest duration of initial symptoms (less than two years) is interesting despite the small group size (extreme value at baseline excluded). All patients in the MCI-AD group were similar in that all were within two years of an AD diagnosis, so the higher level of α-synuclein amongst those with shortest duration since initial symptoms could be indicative of a more rapid disease progression. A similar effect has been observed for tau species in a larger material. Buchhave and colleagues [40] found that early converters from MCI to AD had increased levels of t-tau and p-tau compared to later converters, and suggested that tau biomarkers might reflect disease progression from MCI to AD. If so, such changes related to disease progression might be detectable also in other proteins, such as α-synuclein.

Measurable α-synuclein levels are highly variable between studies, which could be due to many reasons, including small sample sizes, differences in characterization of patient groups, and analytical differences [19]. The present study is relatively small, with large individual variation in the measured α-synuclein levels for all groups, both at baseline (with and without the extreme value), and at follow-up. The ELISA kit used in this study has been shown not only to measure α-synuclein monomers, but also dimers and fragments of similar size [23]. Consequently, variations in the balance of monomers to dimers or other oligomers in CSF between groups would not be observed with this method.

## Conclusion

CSF levels of α-synuclein in a follow-up study of patients with AD or with aMCI divided as to whether patients progressed to AD during the two years of study or not, did not show any significant differences in concentration compared to each other over time, or to a control group at baseline. However, CSF α-synuclein was strongly correlated with Aβ40 (but not Aβ42), and moderately correlated with tau species both among patients and control individuals. Patients in the aMCI subgroup progressing to AD that had a short duration of symptoms by the time of inclusion had significantly higher CSF α-synuclein levels than those with longer duration, but subgroups were small. These results highlight the importance of knowing exactly which species of α-synuclein are being measured by any given kit when comparing CSF levels in different studies. According to our results using the present method, α-synuclein is not a potential biomarker for AD. However, other analyses determining other molecular species of α-synuclein might be more promising.

## Additional files

Additional file 1: Table S1. a) Observed group mean values (± SD) for α-synuclein at inclusion (baseline), and after one and two years, and b) predicted mean value (95 % CI) at baseline, together with estimated mean change (95 % CI) from baseline values.1 (DOCX 14 kb) Additional file 2: Table S2. Mean levels of α-synuclein in CSF at inclusion for carriers and non- carriers of *APOE* ε4. (DOCX 12 kb)

## Abbreviations

Aβ40: Amyloid-β 1–40
Aβ42: Amyloid-β 1–42
AD: Alzheimer’s disease
aMCI: Amnestic mild cognitive impairment
CSF: Cerebrospinal fluid
ELISA: Enzyme-linked immunosorbent assay
LMM: Linear mixed model
MMSE: Mini-mental state examination
p-tau: Phosphorylated tau
t-tau: Total tau
