# Supplementary material for: Alpha-synuclein measured in cerebrospinal fluid from patients with Alzheimer’s disease, mild cognitive impairment, or healthy controls: a two year follow-up study
Source: BMC Neurol. 2016 Sep 21;16:180. doi: 10.1186/s12883-016-0706-0 (PMC5031325; doi:10.1186/s12883-016-0706-0)
Supplement: Additional file 1: Table S1. — a) Observed group mean values (± SD) for α-synuclein at inclusion (baseline), and after one and two years, and b) predicted mean value (95 % CI) at baseline, together with estimated mean change (95 % CI) from baseline values.1 (DOCX 14 kb) [file 12883_2016_706_MOESM1_ESM.docx]

|  | α-synuclein (pg/mL) | | | | | | |  |  |
| --- | --- | --- | --- | --- | --- | --- | --- | --- | --- |
|  | AD-AD | | MCI-AD | MCI-MCI | | CTR | | p-value^2^ | p-value^3^ |
| a) |  | |  |  | |  | |  |  |
| Baseline  1 year  2 years | 494.4 ± 275.0  466.0 ± 324.8  439.4 ± 285.5 | | 775.9 ± 633.0    650.9 ± 458.4  687.2 ± 617.7 | 568.6 ± 390.5  559.5 ± 339.6  565.7 ± 393.3 | | 514.4 ± 263.1  na  na | | 0.08 |  |
| b) |  |  | | |  | |  |  |  |
| Baseline  Mean change  After 1 year  After 2 years  Linear trend^4^ | 479.5 (310.7,648.3)  -13.4  (-141.9, 115.1)  -14.7  (-149.0, 119.6)  -7.4  ( -74.3,59.4) | 768.2  (596.0,940.4)  -125.4  (-258.5, 7.7)  -93.2  (-226.2, 39.9)  -46.6  (-112.8, 19.6) | | | 568.6 (390.5,746.7)  - 9.0  (-143.8, 125.8)  - 0.3  (-137.2, 136.4)  - 0.3  (-68.4, 67.9) | |  | 0.09  0.23  0.19 | 0.72  0.58 |

**Additional file 1: Table a)** Observed group mean values (± SD) for α-synuclein at inclusion (baseline), and after one and two years, and **b)**  predicted mean value (95% CI) at baseline, together with estimated mean change (95% CI) from baseline values.^1^

na = not applicable

^1^ Results based on linear mixed model (LMM) with subject as random factor

^2^ F-test for overall difference in mean α-synuclein levels between study groups (one-way ANOVA), or between patient groups (LMM)

^3^ F-test for heterogeneity in mean change over time in α-synuclein between patient groups (categorical and linear interaction, respectively)

^4^ Mean change per year, based on model with linear time trend
