# Supplementary material for: Alpha-synuclein measured in cerebrospinal fluid from patients with Alzheimer’s disease, mild cognitive impairment, or healthy controls: a two year follow-up study
Source: BMC Neurol. 2016 Sep 21;16:180. doi: 10.1186/s12883-016-0706-0 (PMC5031325; doi:10.1186/s12883-016-0706-0)
Supplement: Additional file 2: Table S2. — Mean levels of α-synuclein in CSF at inclusion for carriers and non- carriers of APOE ε4. (DOCX 12 kb) [file 12883_2016_706_MOESM2_ESM.docx]

| α-synuclein (pg/mL) | | | |
| --- | --- | --- | --- |
|  | ***APOE* ɛ4 carriers**  **Mean (SD)** | ***APOE* ɛ4 non-carriers**  **Mean (SD)** | **p-value^1^** |
| **Total** | 621.5 ± 474.9 | 529.6 ± 336.2 | 0.30 |
| By study group |  |  |  |
| **AD-AD** | 518.3 ± 293.8 | 399.0 ± 171.7 | 0.39 |
| **MCI-AD** | 752.8 ± 406.0* | 442.3 ± 181.1 | 0.14 |
| **MCI-MCI** | 494.3 ± 229.9 | 612.2 ± 525.4 | 0.49 |
| **CONTROLS** | 452.0 ± 249.1 | 538.7 ± 271.3 | 0.43 |
|  |  |  |  |

**Additional file 2 Table .** Mean levels of α-synuclein in CSF at inclusion for carriers and non-carriers of *APOE* ε4

^1^T-test for difference in mean value between *APOE* ε4 carriers and non-carriers.

*If extreme value is included: 887.1 ± 692.9 pg/mL
